# Supplementary material for: Projected Incidence of Hepatobiliary Cancers and Trends Based on Age, Race, and Gender in the United States
Source: Cancers (Basel). 2024 Feb 6;16(4):684. doi: 10.3390/cancers16040684 (PMC10886529; doi:10.3390/cancers16040684)
Supplement: Supplementary file 1 [file cancers-16-00684-s001.zip › cancers-2805936-supplementary.pdf]

Supplementary Materials

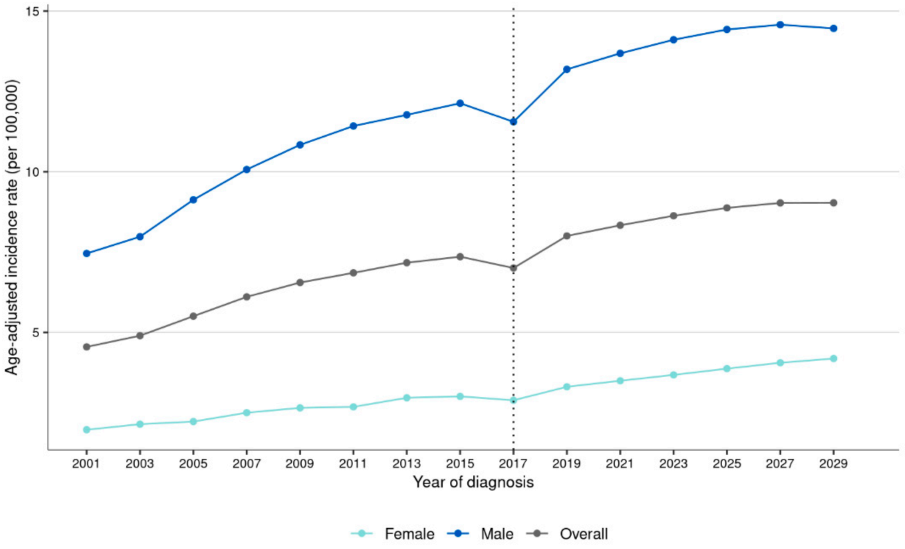

Supplemental Figure S1. Observed (2001-2017) and projected (2018-2029) incidence of hepatocellular carcinoma by sex.

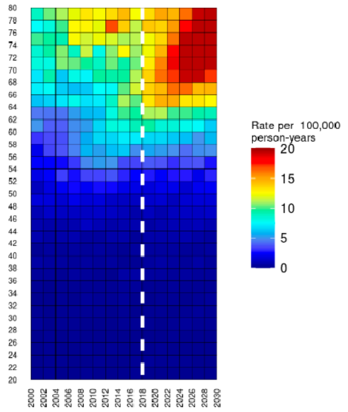

a.

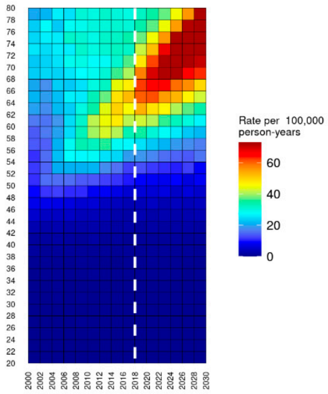

b.

Supplemental Figure S2. Observed (2001-2017) and projected (2018-2029) incidence of hepatocellular carcinoma by age in **(a)** females and **(b)** males.

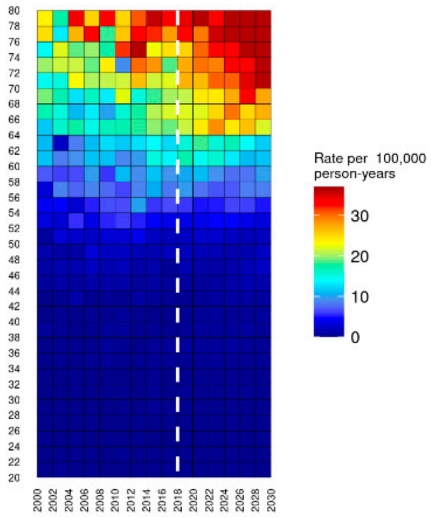
Rate per 100,000 person-years
30
20
10
0
80 78 76 74 72 70 68 66 64 62 60 58 56 54 52 50 48 46 44 42 40 38 36 34 32 30 28 26 24 22 20
2000 2002 2004 2006 2008 2010 2012 2014 2016 2018 2020 2022 2024 2026 2028 2030

a.

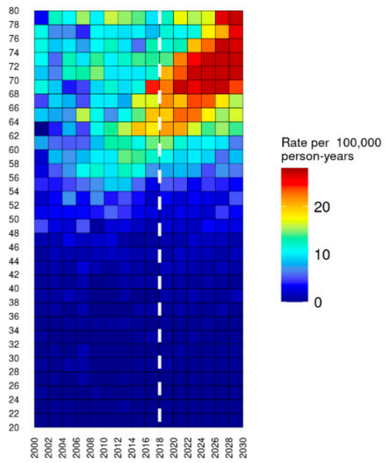
Rate per 100,000 person-years
20
10
0
80 78 76 74 72 70 68 66 64 62 60 58 56 54 52 50 48 46 44 42 40 38 36 34 32 30 28 26 24 22 20
2000 2002 2004 2006 2008 2010 2012 2014 2016 2018 2020 2022 2024 2026 2028 2030

b.

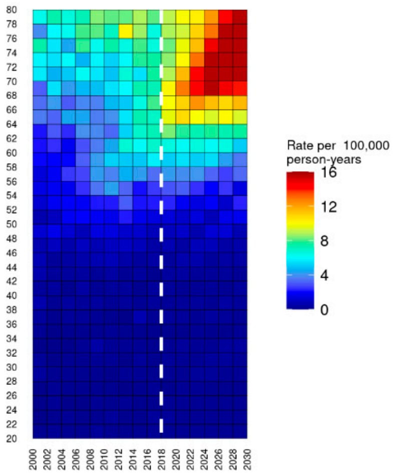
Rate per 100,000 person-years
16
12
8
4
0
80 78 76 74 72 70 68 66 64 62 60 58 56 54 52 50 48 46 44 42 40 38 36 34 32 30 28 26 24 22 20
2000 2002 2004 2006 2008 2010 2012 2014 2016 2018 2020 2022 2024 2026 2028 2030

c.

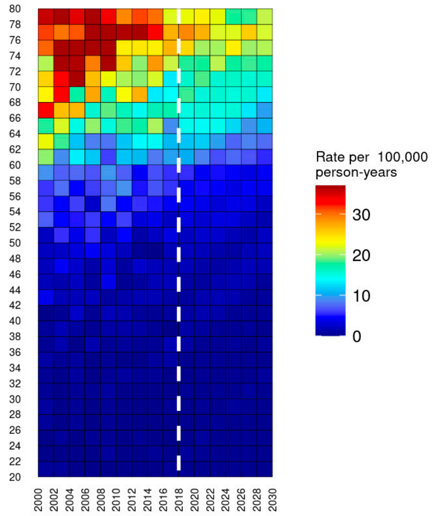

d.

Supplemental Figure S3. Observed (2001-2017) and projected (2018-2029) incidence of hepatocellular carcinoma in (a) Hispanic, (b) Black, (c) White, and (d) Asian females by age.

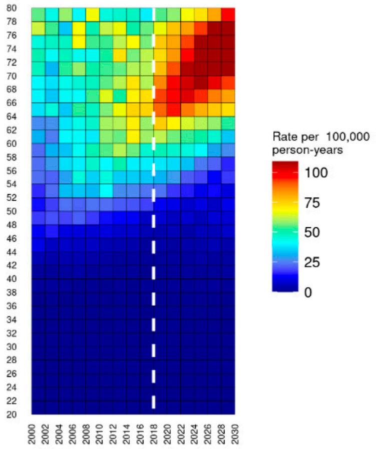

a.

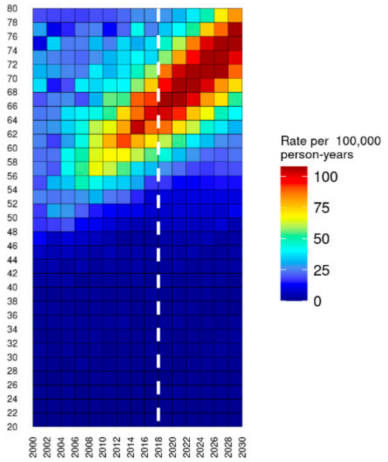

b.

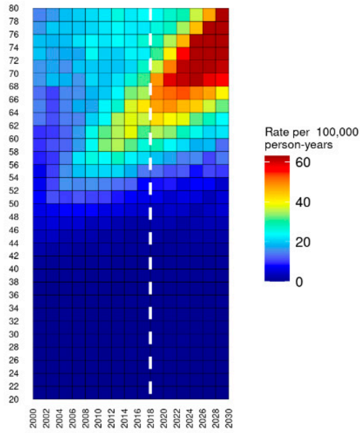

c.

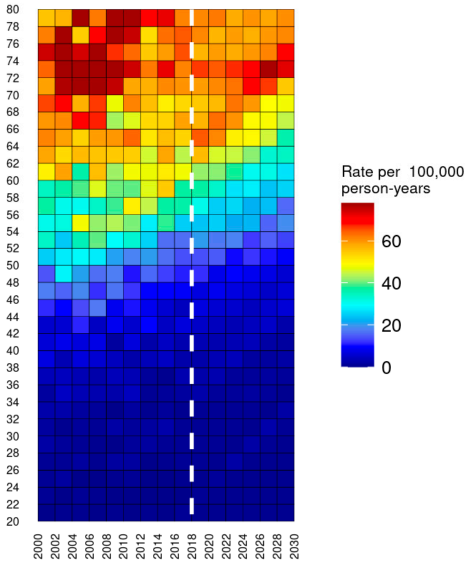

d.

Supplemental Figure S4. Observed (2001-2017) and projected (2018-2029) incidence of hepatocellular carcinoma in (a) Hispanic, (b) Black, (c) White, and (d) Asian males by age.

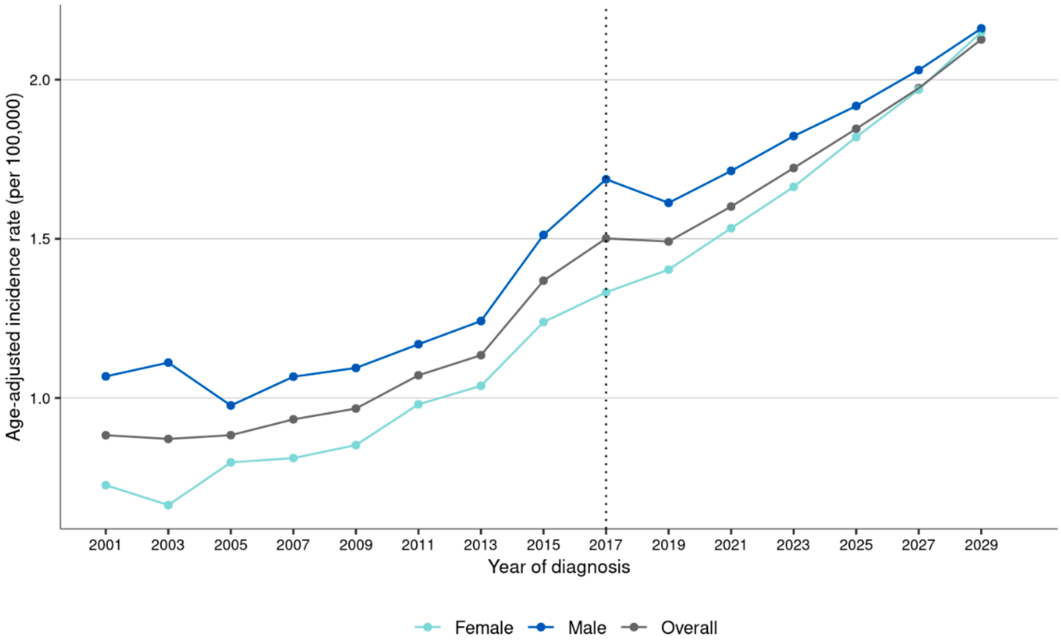

Supplemental Figure S5. Observed (2001-2017) and projected (2018-2029) incidence of intrahepatic cholangiocarcinoma by sex.

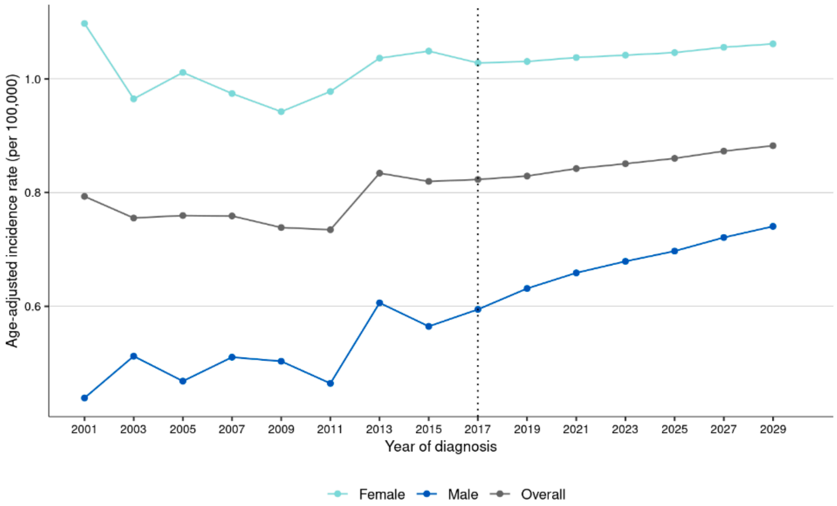

Supplemental Figure S6. Observed (2001-2017) and projected (2018-2029) incidence gallbladder carcinoma by sex.

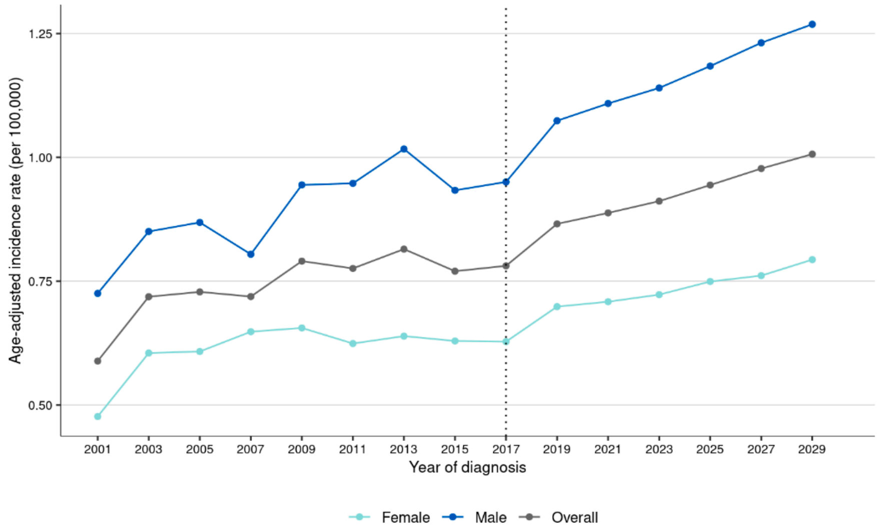

Supplemental Figure S7. Observed (2001-2017) and projected (2018-2029) incidence of extrahepatic cholangiocarcinoma by sex.

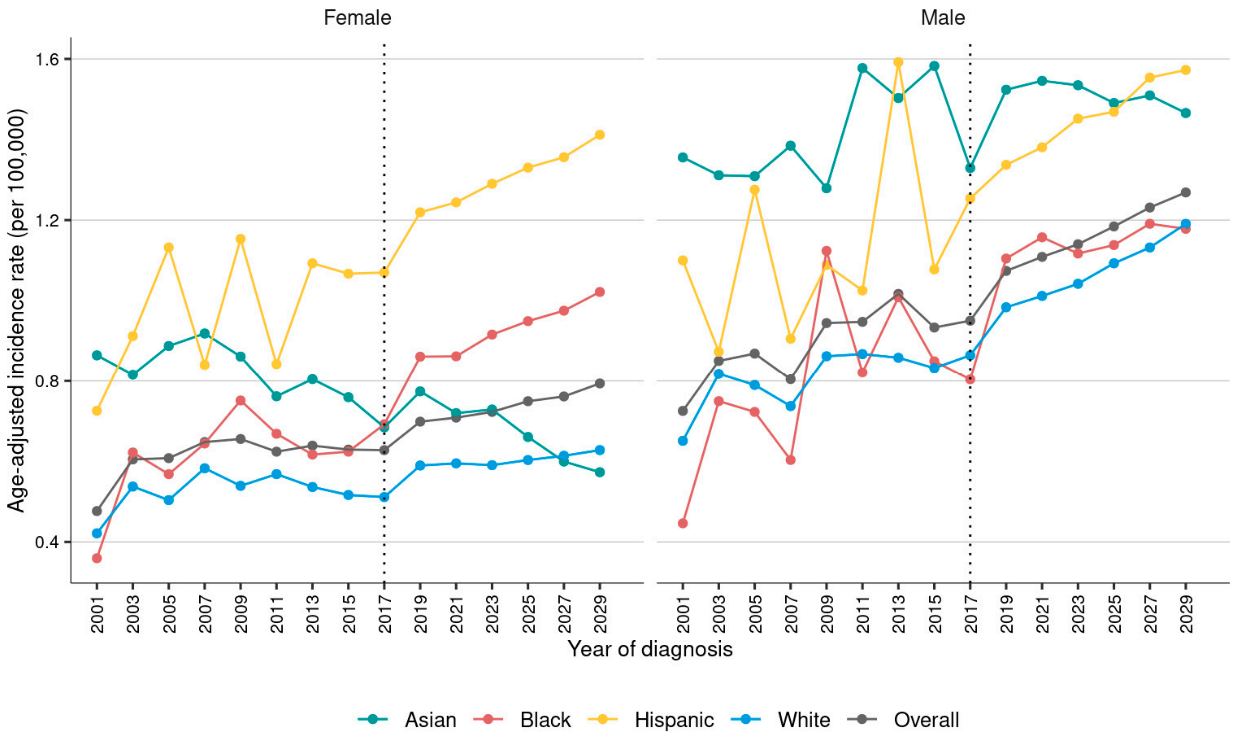

Supplemental Figure S8. Observed (2001-2017) and projected (2018-2029) incidence of extrahepatic cholangiocarcinoma by race in females and males.

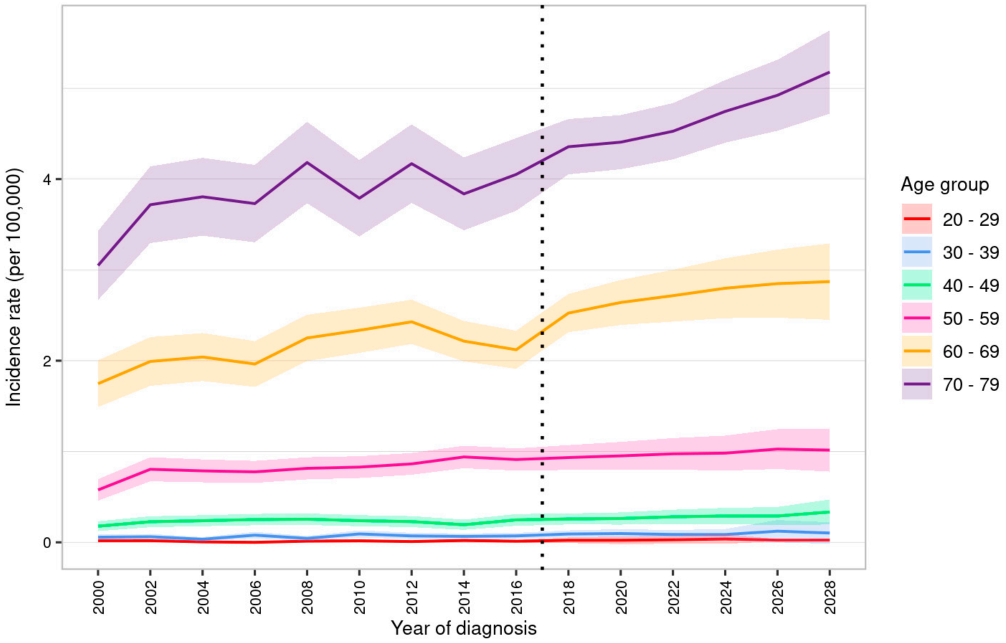

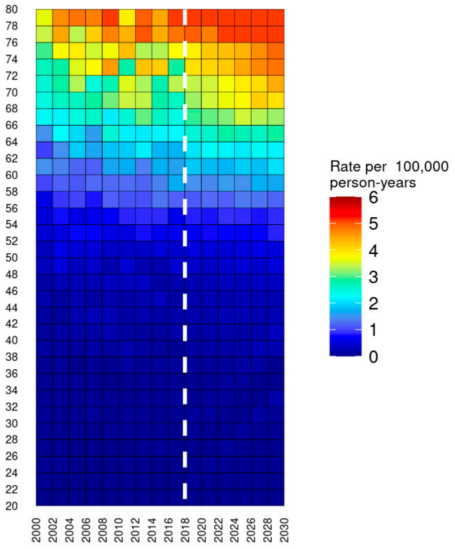

Supplemental Figure S9. Observed (2001-2017) and projected (2018-2029) incidence of gallbladder carcinoma by age via **(a)** graph and **(b)** heat map.

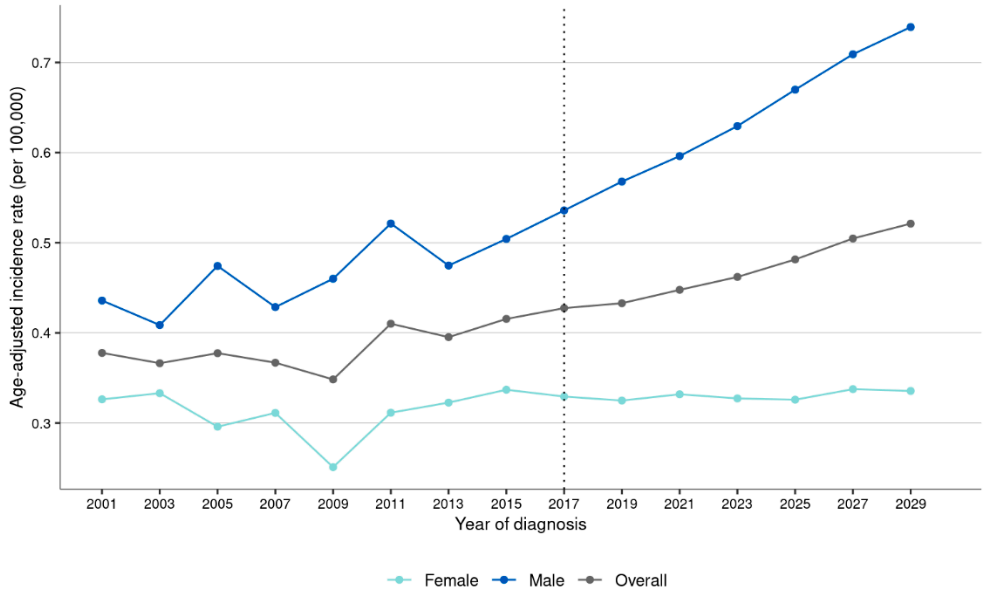

Supplemental Figure S10. Observed (2001-2017) and projected (2018-2029) incidence of ampullary carcinoma by sex.

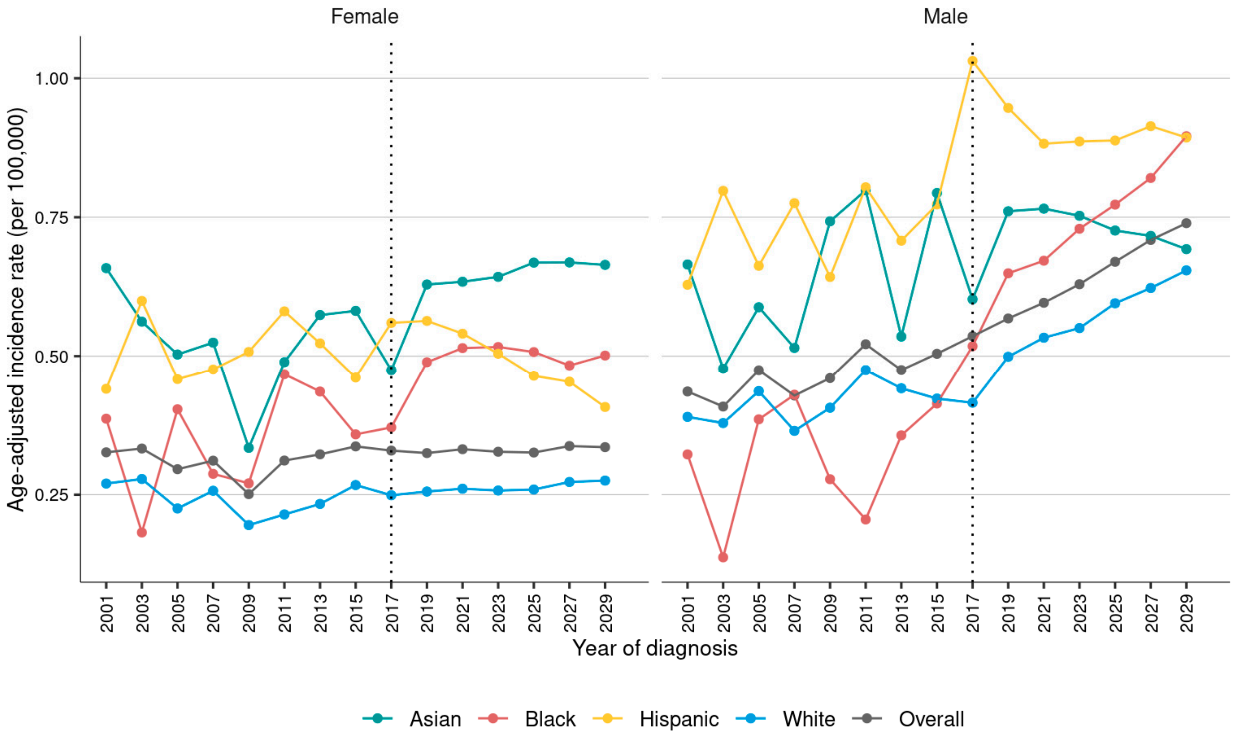

Supplemental Figure S11. Observed (2001-2017) and projected (2018-2029) incidence of ampullary carcinoma by race in females and males.

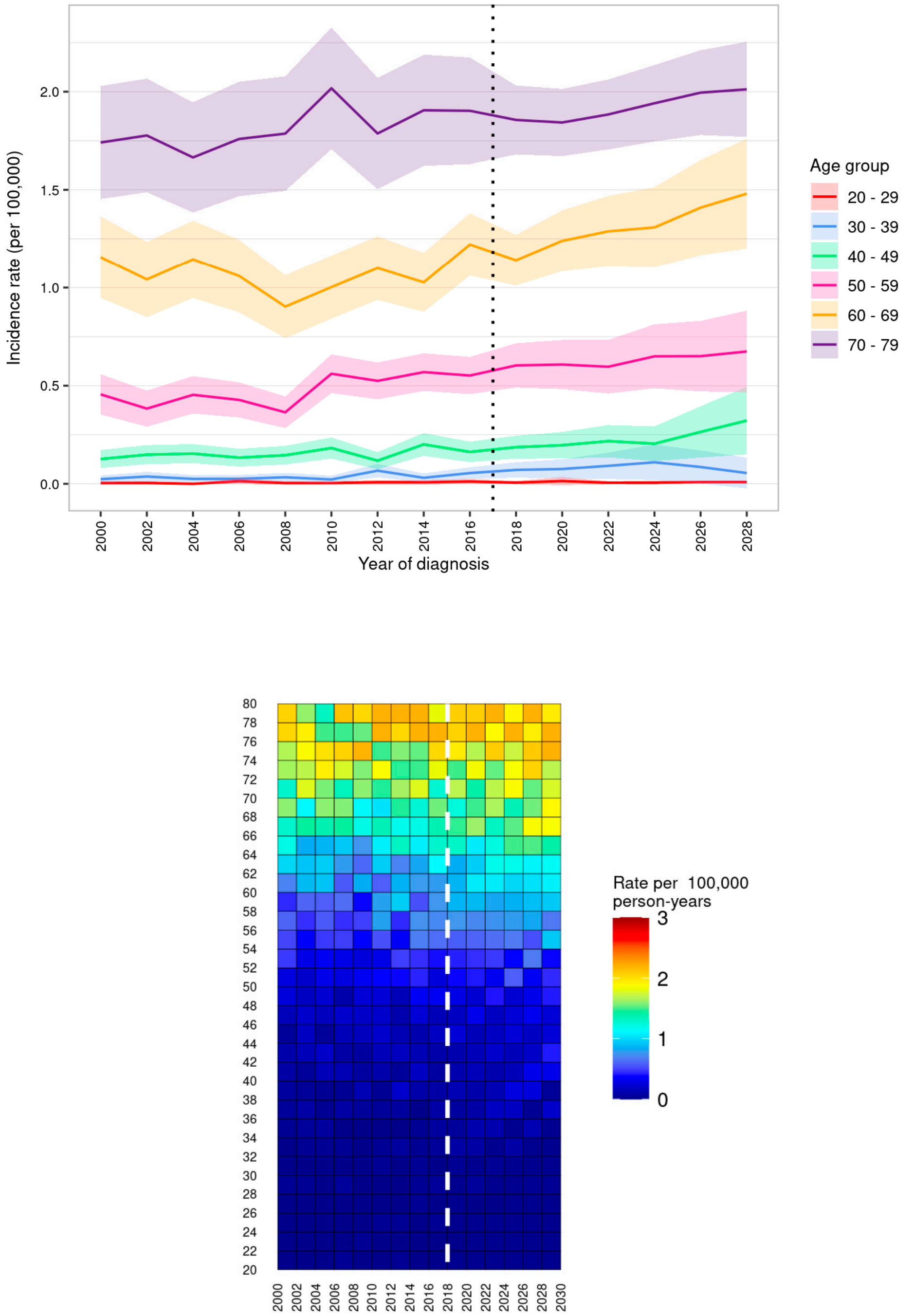

Supplemental Figure S12. Observed (2001-2017) and projected (2018-2029) incidence of ampullary carcinoma by age via **(a)** graph and **(b)** heat map.
